# A Comparative Study of the Applied Methods for Estimating Deflection of the Vertical in Terrestrial Geodetic Measurements

**DOI:** 10.3390/s16040565

**Published:** 2016-04-20

**Authors:** Luca Vittuari, Maria Alessandra Tini, Pierguido Sarti, Eugenio Serantoni, Alessandra Borghi, Monia Negusini, Sébastien Guillaume

**Affiliations:** 1 DICAM—University of Bologna, Viale Risorgimento 2, Bologna 40136, Italy; mariaalessandra.tini@unibo.it; 2 Istituto di Radioastronomia (IRA)—Istituto Nazionale di Astrofisica (INAF) Via P. Gobetti, 101 Bologna 40129, Italy; p.sarti@ira.inaf.it (P.S.); negusini@ira.inaf.it (M.N.); 3 ETH Zurich—Institute of Geodesy and Photogrammetry, Stefano-Franscini-Platz 5, Zurich 8093, Switzerland; eugenio.serantoni@geod.baug.ethz.ch (E.S.); guillaume@geod.baug.ethz.ch (S.G.); 4 University eCampus, via Isimbardi 10, Novedrate, CO 22060, Italy; alessandra.borghi@uniecampus.it

**Keywords:** deflection of the vertical, tie vectors, ITRF, QDaedalus, ITALGEO2005

## Abstract

This paper compares three different methods capable of estimating the deflection of the vertical (DoV): one is based on the joint use of high precision spirit leveling and Global Navigation Satellite Systems (GNSS), a second uses astro-geodetic measurements and the third gravimetric geoid models. The working data sets refer to the geodetic International Terrestrial Reference Frame (ITRF) co-location sites of Medicina (Northern, Italy) and Noto (Sicily), these latter being excellent test beds for our investigations. The measurements were planned and realized to estimate the DoV with a level of precision comparable to the angular accuracy achievable in high precision network measured by modern high-end total stations. The three methods are in excellent agreement, with an operational supremacy of the astro-geodetic method, being faster and more precise than the others. The method that combines leveling and GNSS has slightly larger standard deviations; although well within the 1 arcsec level, which was assumed as threshold. Finally, the geoid model based method, whose 2.5 arcsec standard deviations exceed this threshold, is also statistically consistent with the others and should be used to determine the DoV components where local *ad hoc* measurements are lacking.

## 1. Introduction

Global Navigation Satellite Systems (GNSS) are nowadays extensively used to survey geodetic networks with different levels of precision. However, there are countless applications where satellite systems must be integrated with traditional three-dimensional terrestrial surveying techniques. These include, e.g., underground engineering surveying and any surveying application where equipotential surfaces are relevant, such as in the hydraulic infrastructure framework or during the construction of large instruments, (e.g., large-scale interferometers or particle accelerators, *etc.*), where extended geoidal surfaces are often to be determined. For these applications, global geometric techniques, such as GNSS, must be combined with more traditional observation techniques and instruments (total stations, levels, gyroscopic theodolites, nadir/zenith levels, *etc.*), whose set up and measurements depend on the local plumb line, *i.e.*, on the tangent to the line of force of the terrestrial gravity field at the instrument [1,2,3]. In order to integrate the observations of GNSS and traditional instruments, it is necessary to know the Deflection of the Vertical (DoV), defined as the angle between the plumb line and the ellipsoidal normal in a given point. There are areas where the ellipsoid can considerably differ from the geoid surface, in particular for globally oriented geocentric ellipsoids [4]. The values of the DoV can be as large as several tens of arcseconds [5] and can exhibit significant variations in relation to the change of topography and to the spatial variation of the lithosphere density.

The DoV values and spatial variations can well exceed the precision of the measurements and, often, its impact cannot be ignored when solely terrestrial technique based surveys are also concerned [4,6]. In the 1980s and 1990s when satellite navigation systems became fully accessible for a geodetic use, the development of the adjustment theory to be applied to mixed three-dimensional networks surveyed by GPS and traditional techniques required the inclusion of the DoV values [7,8,9,10,11].

One striking example of the importance of the DoV is related to the alignment of the tie vectors for the combination of space geodetic solutions and the computation of the International Terrestrial Reference Frame (ITRF) [12]. In fact, the ITRF is realized combining the global solutions of GNSS, Very Long Baseline Interferometry (VLBI), Doppler Orbitography and Radiopositioning Integrated by Satellite (DORIS) and Satellite Laser Ranging (SLR) and a key role is played by the co-location sites where two or more geodetic techniques are operated. At these sites, the connection between each single technique frame is realized measuring the tie vector, *i.e.*, the reciprocal position of the reference points of each instrument. The tie vectors are generally measured using classic topographical (traditional) instruments and, therefore, are initially obtained in a local topocentric reference system. In the ITRF computation, the tie vectors must be aligned with the global reference frame before the combination process is realized. The alignment can be performed in various ways and it is always critical, as any misalignment could degrade the intrinsic precision of the tie vector and impact the ITRF solution. A comprehensive discussion about the role of the co-location sites in space geodesy and about the tie vectors and their alignment in the ITRF computation can be found in [13]. 

A common alignment procedure is based on the least-squares method: the tie vector is rotated and translated via a similarity transformation performed using common points, *i.e.*, points surveyed using both terrestrial and GNSS techniques. However, this method might be insufficiently accurate for small networks: the limited accuracy of GNSS-derived ellipsoidal heights will certainly affect the positions of common points and will particularly reflect on the orientation accuracy of the shortest baselines (e.g., a couple of hundred meters or less). 

A particularly effective alternative is represented by the deterministic method described in [14,15], where an initial transformation, based on the knowledge of the DoV, from the local topocentric Cartesian system to the local geodetic Cartesian system is followed by a second transformation, from the latter to the global frame. [16] demonstrated that ignoring the first of the two transformations, and therefore neglecting the DoV, may cause an error of one centimeter on the tie vector components, which is totally incompatible with the mm-level precision required for the ITRF computation [17]. 

The DoV is generally split into two orthogonal components, one in the meridian plane and one in the plane of the prime vertical, denoted *ξ* and *η*, respectively [5]. The total deflection *θ* can then be obtained from:

(1) $$\theta^{2}=\xi^{2}+\eta^{2}$$

The component of DoV, along a geodetic azimuth direction *α*, is given by [18]:

(2) ε=ξ cosα+η sinα

The curvature of the lines of force of gravity causes the value of the DoV to change along the plumb line. There are different definitions of the DoV, depending on where, along the plumb line, it is evaluated [19,20,21,22]. Among these, the definition given by Helmert, *i.e.*, the DoV at the topographical surface (rather than, e.g., at the geoid), yields the value that must be applied for integrating the data acquired by instruments of space geodesy with those acquired by terrestrial instruments.

The components of the DoV at the surface can be simply estimated comparing the astronomical latitude and longitude *(Φ, Λ)* with the ellipsoidal geodetic coordinates *(ϕ, λ)* for the same point (astro-geodetic method) [5],

(3) $$\begin{array}{c} \xi =\Phi -\varphi \\ \eta =\left(\Lambda -\lambda \right)cos\varphi \end{array}$$

In this study, we compare the DoV values that were obtained with three different methods at the two GNSS-VLBI co-location sites of Medicina (Northern Italy) and Noto (Sicily), managed by the Institute of Radioastronomy (IRA) of the National Institute of Astrophysics (INAF).

At both sites, since 2001 the GNSS-VLBI tie vectors have been periodically surveyed using terrestrial techniques and have contributed to the computation of the ITRF [12]. Under this perspective, the knowledge of the DoV might play a crucial role in achieving the utmost level of accuracy in the tie vectors alignment.

In the next section, the three different methods that were adopted to estimate the DoV are illustrated: (i) a combination of GNSS and geometric leveling (GNSS-LEV); (ii) the QDaedalus astro-geodetic system developed by the ETH Zurich [23]; and (iii) the estimation of the DoV components using the most accurate Italian gravimetric geoid model available: ITALGEO2005 [24]. Section 3 is devoted to the discussion of the results and the last section contains the conclusions.

## 2. DoV Estimation: Description of the Methods 

**Figure 1:**
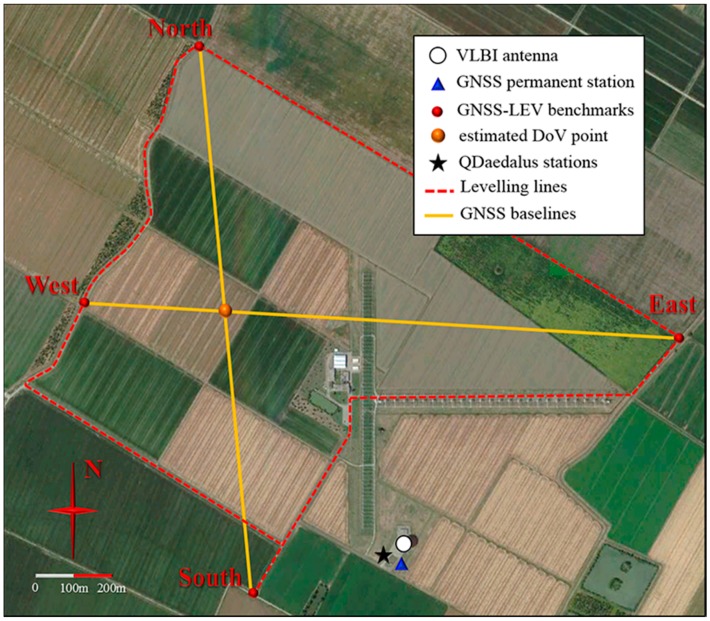
Area of IRA-INAF observatory of Medicina (Northern Italy) (satellite image courtesy of Google Earth^®^).

The Medicina observatory is situated in the Southern portion of the alluvial Po River Plain, where the terrain morphology is characterized by a flat and regular surface (Figure 1). The substrata are equally rather homogeneous both in terms of composition and stratigraphy (e.g., see [16]) and these features reflect into small values and regular variations of the DoV components around the site. The clay and silt content of the top layer of the terrain generates noticeable swelling/constricting phenomena as a function of atmospheric precipitation [25].

**Figure 2:**
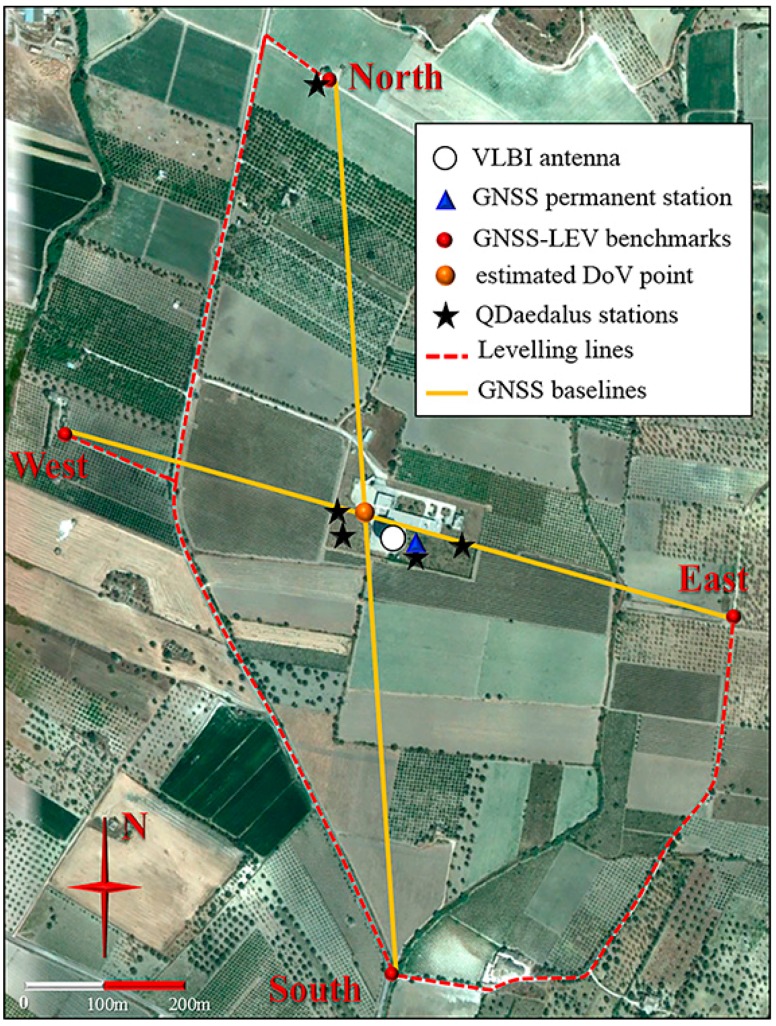
Area of the IRA-INAF observatory of Noto (Sicily) (satellite image courtesy of Google Earth^®^).

The Noto observatory is characterized by a radically different geomorphology (Figure 2). It is located in the Northern edge of the Tellaro River Plain and is characterized by Quaternary alluvial deposits accumulated during the Pleistocene [26]. Locally, a hill range rises to a few hundred meters and surrounds the Eastern and Northern part of the site. The proximity to the boundary between the African and the Eurasian plates, together with the irregular surface morphology, induces inhomogeneous variations in the geoid surface.

### 2.1. Determination of the DoV Using the GNSS-LEV Technique 

**Figure 3:**
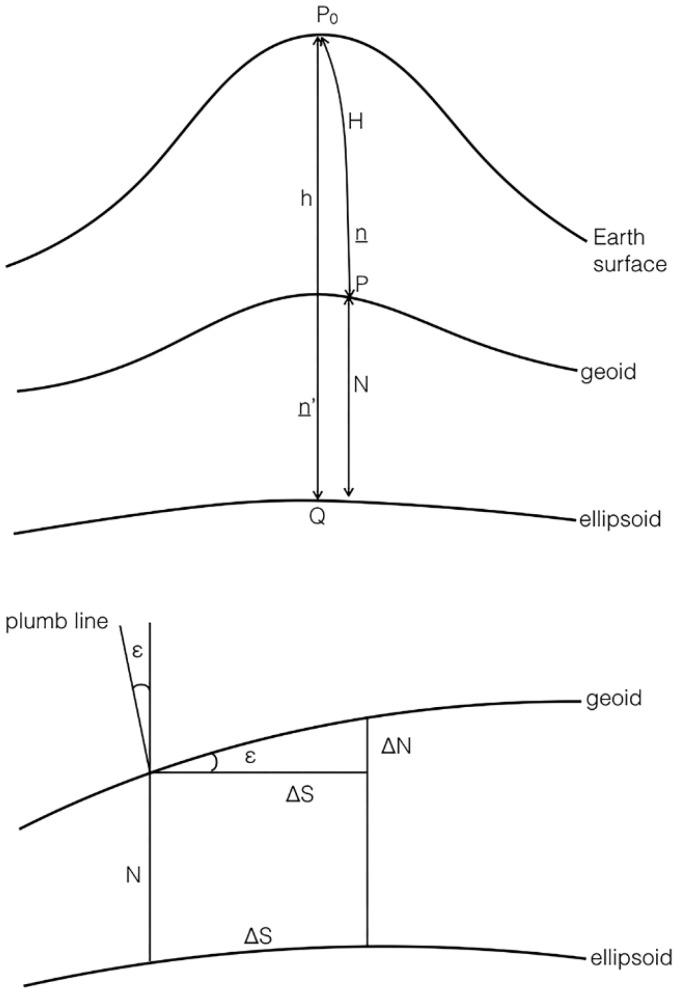
Relation between the geoid undulation N and the deflection of the vertical *ε*. (Adapted from [27]).

Geoid undulation and DoV values can be estimated with the combined use of GNSS techniques and geometric leveling. The theoretical foundation of the method is based on the relation [27]:

(4) $$\varepsilon \approx -\frac{\Delta N}{\Delta S}=-\frac{\Delta h-\Delta H}{\Delta S}$$

where *ε* is the DoV value in the direction of the GNSS baseline, Δ*S* is the length of the geodesic line and Δ*N* is calculated by the difference between the ellipsoidal height difference (Δ*h*), measured using GNSS, and the height difference (Δ*H*) obtained from geometric leveling (Figure 3). Combining Equation (4) with Equation (2), we get:

(5) $$-\frac{\Delta N}{\Delta S}\approx \xi \;cos\alpha +\eta \;sin\alpha$$

where *α* is the ellipsoidal azimuth of the GNSS baseline and Δ*S* is the length of the corresponding geodesic arc. Measuring the geoidal and the ellipsoidic height differences in, at least, two directions, the two unknown components of the DoV can be calculated using Equation (5).

The topographic surveys were planned to estimate the components of the DoV with a precision comparable to that of the angular readings of high-end total stations, *i.e.*, ≈ 0.5 arcsec. Nevertheless, this level of precision is hardly preserved, even when high precision accessories and refined operative arrangements are adopted. A 1.5 arcsec accuracy level is more likely achieved under real operational conditions, particularly on networks of limited size. Considering that, as a rule of thumb, the precision on DoV estimate can affect the angle measurement standard deviation up to 40%, we fixed a threshold of 1 arcsec as maximum uncertainty on the DoV estimate. This threshold is compatible with an overall angular readings standard deviation lower than 2 arcsec.

Assuming a standard deviation of 3 mm in the determination of the height differences on short baselines measured with static GNSS surveying (using high-quality antennas and state-of-the-art scientific software for post-processing) and a standard deviation of 1 mm/√km for geometric leveling, the desired 1 arcsec DoV standard deviation over a 1 km baseline can be obtained. Therefore, two approximately orthogonal GNSS baselines with lengths varying between 0.9 and 1.3 km were measured at both observatories. Within this distance range, the DoV value *ε* can be referred to a unique average value along the baseline [27]. Following this approach, we estimated the DoV components ξ and η with respect to the point of intersection between the two GNSS baselines, as shown in Figure 1 and Figure 2. These values, although not being determined at the exact position of the tie vector, can be easily referred to it, with a good approximation: the geoid model at the two sites varies by 0.1–0.2 arcsec over the baseline, a variation which is much smaller than our working precision and that can therefore be disregarded.

**Figure 4:**
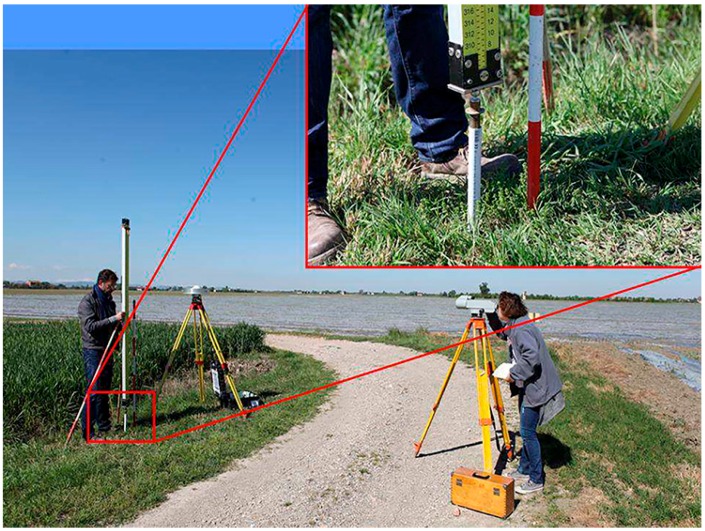
Zeiss Ni1 analogic measurement of the height difference between GNSS-ARP and the South leveling bolt at site Medicina. The inset shows the reference height marker: a bolt on top of a 1.5 m coated metal rod hammered into the ground.

At Medicina observatory, the measurements were carried out in March 2014. In Figure 1, the four endpoints of the GNSS baselines are identified by red dots. They were actually materialized with a metallic hemispherical nail head. Due to the lack of stable manmade objects (having sufficiently deep foundations and being suitable for installing the nails) nearby, the East and South endpoints for the leveling markers were materialized with leveling bolts screwed on top of metal rods (see the inset of Figure 4). The rods were hammered down to a depth of about 1.5 m, this being sufficient to avoid short term height changes caused by the plastic variations of the silty clay cohesive soil in response to precipitation [25]. In addition, the top 1 m portion of the iron rods was isolated and protected from soil suction using a plastic tube coating (the white tube visible in the inset of Figure 4). 

The closed leveling loop shown in Figure 1 has an approximate length of 4.8 km and was measured with high precision spirit leveling in one day by two teams, using the digital levels Trimble DiNi12 and Leica DNA03 (first class: 0.3 mm/km). The different sections of the loop were measured forward and backward obtaining section closing errors well within the 1 mm/km limit overall sections. Performing a proper error propagation, a final 0.9 mm closure error was determined over the entire loop. 

At Noto observatory, the measurements were carried out in July 2014. The firmer terrain conditions and the availability of stable manmade artifacts made the use of metal rods to materialize the GNSS baseline endpoints unnecessary. However, no path directly connecting the East and North endpoints could be found. As a consequence, the high precision spirit leveling could not be carried out in a closed loop. The total length of the leveling loop amounts approximately to 2.4 km. The loop was measured forward and backward and it is represented in Figure 2. The measurements were carried out in two days by only one surveying team equipped with a Leica DNA03 digital level. The maximum discrepancy recorded between forward and backward measurements was 0.6 mm over a 600 m section, which is well compatible with the reference standard deviation of 1 mm/km for leveled height differences. 

Medicina and Noto leveling data sets were processed using STAR*NET software [28]. 

As for the GNSS surveys, at both sites, 8 h static sessions were carried out during the leveling surveys, using Trimble 5700 receivers and Leica AT504 choke ring antennas. The data were processed using Bernese GPS software [29]. The RMS errors obtained for the Up component downstream of data processing are in the range of 0.2 to 0.4 mm 95% for both sites. We consider these estimates too optimistic and therefore we prudentially account the above-mentioned uncertainties on GNSS-derived height differences.

The GNSS antennas were set up in close proximity to the baseline endpoints (at a distance of less than 2 m). The height difference between the GNSS Antenna Reference Point (ARP) and the leveling benchmark was measured with a first class Zeiss Ni1 analogical level, which was set up at the same height of the ARP in the operating range of the micrometer level (Figure 4).

### 2.2. Determination of DoV Using the QDaedalus System

The DoV components were also measured using QDaedalus, an instrument designed and built at the Institute of Geodesy and Photogrammetry of ETH Zurich [23].

QDaedalus consists of a CCD camera clipped on a total station, in our case a Leica TCA2003 (σ = 0.5 arcsec), in replacement of the eye-piece, a pluggable front lens, a low cost u-blox LEA-6T GNSS receiver, and a laptop running the dedicated software for steering, imaging and processing [30].

The instrument is able to automatically search the most relevant stars thanks to the internal star catalogue (FK6) [31] and it measures the local star vector by means of the embedded image recognition algorithm. For astro-geodetic applications, the centroid of the image of the stars is detected and tracked. The observation equation underlying the determination of the astronomical coordinates is the following [32]:

(6) $$x_{topo}\left(t\right)=\;T\left(\Lambda ,\Phi \right)\;x_{ITRS\;}\left(t\right)=\;T\left(\Lambda ,\Phi \right)\;R_{2}\left(-x_{p}\right)\;R_{1}\left(-y_{p}\right)\;R_{3}\left(GAST\right)\;N\left(t\right)\;P\left(t\right)\;x_{ICRS*\;}\left(t\right)$$

where:

$x_{\text{topo}}\left(t\right)$ = star vector in the local topocentric Cartesian system;
$x_{\text{ITRS}}\left(t\right)$ = star vector in ITRS (International Terrestrial Reference System);
$x_{\text{ICRS*}}\left(t\right)$ = star vector in ICRS (International Celestial Reference System) corrected for parallax, relativity and aberration;
T(Λ,Φ) = transformation matrix from ITRS to the local topocentric system;
$R_{2}\left(-x_{p}\right)\;R_{1}\left(-y_{p}\right)$ = polar motion matrices;
$R_{3}\left(\text{GAST}\right)\;$= Earth rotation matrix;
N(t) = nutation matrix;
P(t) = precession matrix; and
t = time of the observation.

The star vector in ICRS is computed by means of NOVAS-C routines [33], while the parameters determining the matrices **R_1_**, **R_2_**, **R_3_**, and **N** are retrieved from the IERS Bulletin A [34].

The astronomical coordinates (Λ,Φ) are related to the ITRS ellipsoidal coordinates (λ,φ) measured with GNSS by the deflection of the vertical (η,ξ) according to Equation (3).

The measurements were performed during nights with clear sky. The system allows to reach an accuracy in the order of 0.2–0.3 arcsec [30], and it depends on the number, the distribution and the apparent magnitude of the visible stars. 

In July 2014, the instrument was mounted on some existing pillars near the VLBI and permanent IGS-GNSS antennas of Medicina and Noto sites (see Figure 1 and Figure 2), whose ellipsoidal coordinates were previously determined using geodetic GNSS receivers operated in static mode. Hence, the GPS receiver of QDaedalus was used for timing purposes only. 

When setting up at a new station, the approximate orientation of the instrument has to be established manually, by pointing to a known star such as Polaris.

The robust adjustment of each set of measurements was directly performed in the field, enabling an immediate preliminary control of the results and avoiding gross errors in the instrumental set up and calibration. 

### 2.3. Determination of DoV from the ITALGEO2005 Model

The *ξ* and *η* components of the DoV can be also obtained by gravity measurements, for instance using the approaches described in [20,21], based on high resolution Global Geopotential Model (GGM). However, in the interested area (Noto and Medicina), the authors have available the residual gravity anomaly dataset Δ*g_r_* used for the estimate of the current Italian quasi-geoid model, ITALGEO2005 ([24,35]), whereby these high resolution observations, based on terrestrial gravity measures, have been used for the gravimetric estimate of the deflection of the vertical.

The residual gravity anomalies Δ*g_r_* have been obtained by the ITALGEO2005 authors removing the long wavelength of the gravity field Δ*g_GGM_*, predicted by a Global Geopotential Model and the high frequency component Δ*g_RTC_*, due to the gravity effect of the masses, which are between the actual surface and the surface accounted by the GGM used, that is the so-called Residual Terrain Correction (RTC).

In physical geodesy, using the Least-squares Collocation approach, any linear functional of the anomalous potential T can be estimated if observed values are available, which are themselves linear functionals of T [36]. Therefore, knowing the residual gravity anomalies Δ*g_r_*, which are related to the anomalous potential *T* by the fundamental equation of physical geodesy [27] by the following equation

(7) $$\Delta g=-\frac{\partial T}{\partial n^{\prime }}+\frac{1}{\gamma }\frac{\partial \gamma }{\partial n'}T$$

instead of computing the geoid undulation values, the components of the DoV can be obtained, because the following equation exist

(8) $$\begin{array}{c} \xi =-\frac{1}{\gamma \cdot r}\frac{\partial T}{\partial \varphi }\\ \eta =-\frac{1}{\gamma \cdot r\cdot cos\varphi }\frac{\partial T}{\partial \lambda } \end{array}$$

where *γ* is the normal gravity, *r* is the Earth radius and *n’* the normal line of the reference ellipsoid and Equation (8) represents the spherical approximation [37]. 

Hence, as a further product of the ITALGEO2005 model, we can estimate the vertical deflection components *(ξ, η)*. The quasi-geoid ITALGEO2005 model has been performed using the remove-restore approach with Least-Square Collocation [38], so starting from the results of the ITALGEO2005 remove phase, that is the gravity residuals Δ*g_r_*, the residual vertical deflection components ${\left(\xi ,\eta \right)}_{r}\;$were obtained using the Fast Collocation approach [39]. The restore phase was performed adding the long-wavelength component in term of deflection of the vertical ${\left(\xi ,\eta \right)}_{GGM}\;$and the high frequency ${\left(\xi ,\eta \right)}_{RTC}\;$due to the topography to the residual values of *ξ* and *η*. This procedure can be summarized as follows:

(9) $$\begin{array}{l} \mathrm{Remove}:\;\Delta g_{oss}-\Delta g_{GGM}-\Delta g_{RTC}=\Delta g_{r}\\ S\left(\Delta g_{r}\right)={\left(\xi ,\eta \right)}_{r},\;\text{where S(·) is a solution operator, e.g., Least Squares Collocation}\\ \mathrm{Restore}:\;{\left(\xi ,\eta \right)}_{r}+{\left(\xi ,\eta \right)}_{GGM}+{\left(\xi ,\eta \right)}_{RTC}=\left(\xi_{Ital},\eta_{Ital}\right) \end{array}$$

As the gravity residuals$\;\Delta g_{r}$ of ITALGEO2005 have been obtained removing the long-wavelength signals, $\Delta g_{GGM}$ predicted using the Global Geopotential Model GPM98CR up to degree 720 [40], we are forced to use the same model in the restore phase, although this GGM is outdated, otherwise distortions could be introduced in the data, due to not coherence between models. For the aim of this work, where three different geodetic methodologies for the estimate of the deflection of the vertical are compared, we will demonstrate (next sections) that this updated model is sufficient, but we are confident that next Italian geoid model (under processing, private communication with Prof. Barzaghi), based on one of the new GGM will give better performance.

According to the procedure used for the computation of the residual gravity anomalies ITALGEO2005 [24,35], the *(ξ, η)_RTC_* were computed using the TC program of the GRAVSOFT package [38]. Therefore, the RTC effect in term of DoV components was evaluated modeling by prisms the topography between the Italian Digital Terrain Model, based on the SRTM3 data, and a reference surface, obtained filtering the DTM with a moving average window sized of 10’ × 10’, estimated in the ITALGEO2005 project. The RTC has been evaluated up to a 120 km from each computation point.

**Figure 5:**
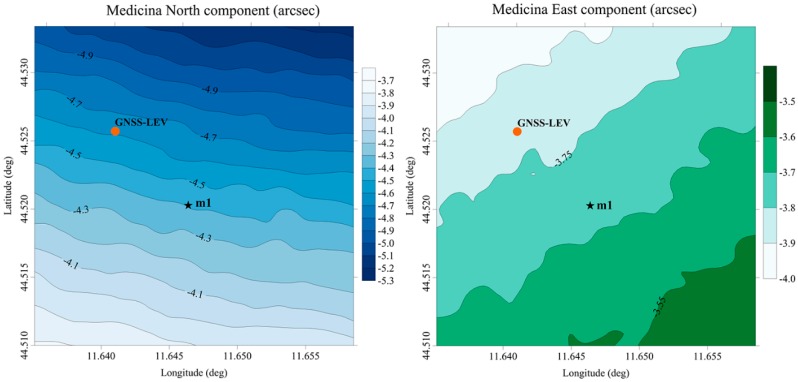
Deflection of the vertical (DoV) components *ξ* (North) and *η* (East) calculated from the ITALGEO2005 grid at the Medicina site. The orange dot indicates the point where the DoV was estimated using the GNSS-LEV method; the black star identifies the location of the DoV measured using QDaedalus.

**Figure 6:**
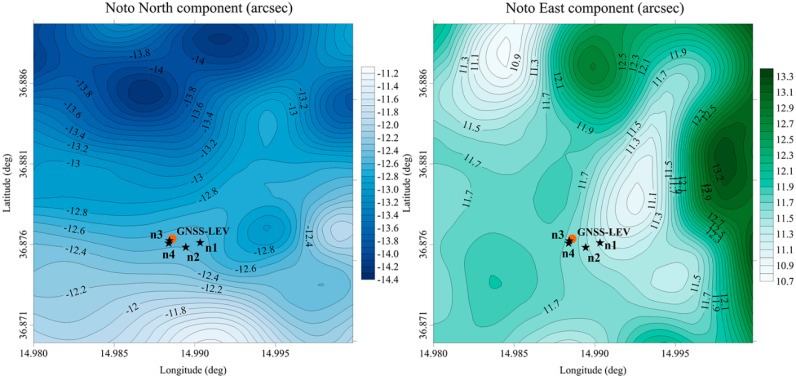
DoV components *ξ* (North) and *η* (East) calculated from the ITALGEO2005 grid at Noto site. The orange dots indicate the point for which the DoV was estimated using the GNSS-LEV method; the black stars identify the locations where the DoV was measured using QDaedalus.

The DoV values obtained from the ITALGEO2005 were predicted over two regular square grids with spacing of 3.24 arcsec covering an area of 3 km^2^ around the sites (see Figure 5 and Figure 6).

## 3. Results and Discussion

The DoV components obtained by the three methods are presented in Table 1 and Table 2 for the sites of Medicina and Noto, respectively. 

For the GNSS-LEV method, the data required for the application of Equation (5) and resulting directly from the GNSS and high precision spirit leveling observations described in Section 2.1 are given in Table S1 (Supplementary Materials). The standard deviations of the *(ξ, η)* components were estimated by variance propagation as described in [41,42].

In the QDaedalus survey, several sets of measurements were taken at every station over a 2 h period. The observation of each of the stars was repeated at least five times. Overall, each set of measurements comprised at least 180 observations. First, each set was adjusted individually (see Table S2 in Supplementary Materials); then, the final values were computed weighting the results of each adjustment according to the respective a-posteriori variance. The standard deviation of the averaged components was less than 0.5 arcsec for every station. 

In order to make direct comparisons, the grid of ITALGEO2005 was examined both at the station points of the QDaedalus system and at the intersection points of the measurement directions of the GNSS-LEV method (see Figure 5 and Figure 6).

In order to compare the two kinds of DoV obtained by the three techniques (geometric and gravimetric, it would be necessary to consider the curvature of the plumb line. The corrections calculated by the formula reported in [27] for the two sites are, respectively, 0.01 arcsec in Noto and 0.002 arcsec in Medicina, negligible with respect to the accuracy of ITALGEO2005 model.

Although to a different level of precision, the three independent methods show, overall, an excellent agreement.

Our DoV estimates also confirm what could be expected from an analysis of the geo-morphological features at the two sites. At Medicina site, low values of the DoV components were found. Differently, at Noto site, the DoV components were found to be more than twice larger over the entire area. This is consistent with what could be expected from the topographical surface features and the tectonic setting of the site areas. The ITALGEO2005 DoV contour maps (Figure 5 and Figure 6) also confirm a smoother and homogeneous variability for Medicina site and rather abrupt variations at Noto site; again, these results are consistent with the sites’ geo-morphological properties.

According to the standard deviations reported in Table 1 and Table 2, the precision of the GNSS-LEV method and the QDaedalus system satisfied the 1-arcsec precision threshold, the latter having the best performances in terms of precision. Conversely, the standard deviations of ITALGEO2005 are larger (2.5 arcsec) and, thus, greater than the limit required for high precision purposes. The ITALGEO2005 standard deviations were deduced via a comparison with astronomical measurements performed by the Italian I.G.M. (Istituto Geografico Militare) over a set of points distributed over the country (R. Barzaghi, private communication). However, in the future, the estimates of the DoV might be improved using the last satellite-based gravimetric missions, e.g., CHAllenging Minisatellite Payload (CHAMP), Gravity Recovery and Climate Experiment (GRACE) and Gravity field and steady-state Ocean Circulation Explorer (GOCE) [43,44,45], thus also improving the performance of the gravimetric method that might currently be jeopardized by the use of the outdated GGM GPM98CR.

The maximum discrepancy between the three methods was found at site Medicina, where ITALGEO2005 underestimates the η component by 1.8 arcsec. Nevertheless, this difference is statistically not significant and does not degrade the excellent consistency of the results.

Although characterized by a lower level of precision, the ITALGEO2005 DoV values should be used in absence of specific DoV measurements. Neglecting the DoV values may result in estimates characterized by a low level of accuracy. 

DoV values are surely important for the alignment of the tie vectors to the ITRF. Lack of information might have effects on the quality of the combined frame. In this case, the DoV values must be measured once, stored and used any time a new tie vector is determined.

Finally, it is worth highlighting that the QDaedalus system not only resulted as the most precise but also as the fastest among the three methods. As stated before, the time required to complete a set of observations at each station (*i.e.*, to obtain DoV component estimates) did not exceed two hours. Cost and time consumption are important parameters against which modern surveying methods must be evaluated.

## 4. Conclusions/Outlook

We compared three different methods for estimating the DoV components at two ITRF sites hosting a VLBI-GNSS co-location. There, the knowledge of the DoV components plays a fundamental role to align the tie vectors measured using terrestrial geodetic instruments to the ITRF. 

The GNSS-LEV method and the QDaedalus system gave results with similar high precision levels. In both cases, the standard deviations of the estimated DoV components meet the 1-arcsec threshold and can be implemented in classic networks without compromising the precision of angular measurements, even with high precision instruments and procedures.

The astro-geodetic measurement using the QDaedalus system has significant advantages: first of all, it allows further improving its precision by greater redundancy and longer observation periods, while being decidedly less onerous than the GNSS-LEV method, from the operational point of view. Furthermore, QDaedalus can measure the DoV components in any desired position, with no need of *a priori* assumptions about the spatial trends of the DoV, as required by the GNSS-LEV method. Conversely, *ad hoc* special equipment is necessary that is still not widely available on the market.

Indeed, almost every surveying company owns the equipment that is required for the GNSS-LEV method, but it is always considerably more onerous in terms of time and staff involved. In addition, site dependent features, such as quality, composition and shape of the topographic surface, may limit or prevent its efficient use, especially when high level of precision must be achieved. 

The ITALGEO2005 grid values showed statistically significant consistencies with the other two methods but associated to standard deviations exceeding the 1-arcsec threshold required to satisfy the angular precision of high precision local networks. Nevertheless, in absence of local DoV measurements, the use of a reliable regional geoid model is recommended anyway.

It is also worth stressing that the use of regional geoid models is promising as their precision is constantly increasing worldwide, thanks to the investigations carried out at a global level by different satellite-based gravity missions, e.g., CHAMP, GRACE and GOCE.

## Figures and Tables

**Table 1:** Components of the DoV obtained by the three methods for the Medicina site; standard deviations are given in brackets.

Medicina	GNSS-LEV		QDaedalus		ITALGEO2005	
	*ξ* (arcsec)	*η* (arcsec)	*ξ* (arcsec)	*η* (arcsec)	*ξ* (arcsec)	*η* (arcsec)
GNSS-LEV point	−5.4 (0.5)	−2.0 (0.5)			−4.6 (2.5)	−3.8 (2.5)
m1			−5.0 (0.1)	−2.0 (0.1)	−4.4 (2.5)	−3.7 (2.5)

**Table 2:** Components of the DoV obtained by the three methods for the Noto site; standard deviations are given in brackets.

Noto	GNSS-LEV		QDaedalus		ITALGEO2005	
	*ξ* (arcsec)	*η* (arcsec)	*ξ* (arcsec)	*η* (arcsec)	*ξ* (arcsec)	*η* (arcsec)
GNSS-LEV point	−12.4 (0.6)	11.1 (0.7)			−12.6 (2.5)	11.7 (2.5)
n1			−11.9 (0.4)	11.8 (0.1)	−12.6 (2.5)	11.4 (2.5)
n2			−13.3 (0.3)	11.5 (0.4)	−12.5 (2.5)	11.5 (2.5)
n3			−12.1 (0.1)	11.9 (0.0)	−12.5 (2.5)	11.7 (2.5)
n4			−12.3 (0.1)	12.2 (0.2)	−12.5 (2.5)	11.7 (2.5)

## Supplementary Materials

Table S1: Measurements and derived values according to Section 2.1. The respective standard deviation is given in brackets. Table S2: Results of the adjustment of the individual sets of measurements obtained from QDaedalus.
