# Supplementary material for: A Comparative Study of the Applied Methods for Estimating Deflection of the Vertical in Terrestrial Geodetic Measurements
Source: Sensors (Basel). 2016 Apr 20;16(4):565. doi: 10.3390/s16040565 (PMC4851079; doi:10.3390/s16040565)
Supplement: Supplementary file 1 [file sensors-16-00565-s001.pdf]

Supplementary Materials: A Comparative Study of the Applied Methods for Estimating Deflection of the Vertical in Terrestrial Geodetic Measurements

Luca Vittuari, Maria Alessandra Tini, Pierguido Sarti, Eugenio Serantoni, Alessandra Borghi, Monia Negusini and Sébastien Guillaume

Table S1. Measurements and derived values according to Section 2.1. The respective standard deviation is given in brackets.

Site	Baseline	Δh (m)	ΔH (m)	ΔN (m)	ΔS (m)	α (deg)
Medicina	S-N	0.7972 (1.2E−03)	0.8317 (3.0E−03)	−0.0345	1339.6 (1.0E−03)	356.51008 (4.3E−05)
	W-E	1.3632 (1.2E−03)	1.3753 (3.0E−03)	−0.0121	1485.5 (1.0E−03)	93.05774 (3.9E−05)
Noto	S-N	−18.9243 (1.1E−03)	−18.8410 (3.0E−03)	−0.0833	1192.7 (1.0E−03)	348.11478 (4.8E−05)
	W-E	0.6467 (1.0E−03)	0.7093 (3.0E−03)	−0.0626	919.4 (1.0E−03)	105.52558 (6.2E−05)

Table S2. Results of the adjustment of the individual sets of measurements obtained from QDaedalus.

Site	Pillar	Set	ξ (arcsec)	η (arcsec)	σ(ξ) (arcsec)	σ(η) (arcsec)	# obs.
Medicina	m1	1	−5.25	−1.77	0.16	0.21	462
		2	−4.73	−1.81	0.1	0.14	208
		3	−5.22	−2.23	0.09	0.12	316
		4	−4.96	−2.14	0.05	0.06	328
Noto	n1	1	−12.15	11.93	0.14	0.17	282
		2	−12.43	11.8	0.12	0.21	576
		3	−11.1	11.76	0.17	0.21	338
	n2	1	−12.81	12.09	0.11	0.13	224
		2	−13.67	11.65	0.12	0.14	252
		3	−13.44	10.6	0.13	0.17	234
	n3	1	−12.23	11.85	0.08	0.09	214
		2	−12.16	11.85	0.07	0.08	252
		3	−12.01	12	0.08	0.09	206
	n4	1	−12.47	12.52	0.08	0.09	256
		2	−12.24	11.91	0.07	0.08	188
		3	−12.06	12.16	0.07	0.07	290
